# Treatment decision for transcatheter aortic valve implantation: the role of the heart team: Position statement paper of the Dutch Working Group of Transcatheter Heart Interventions

**DOI:** 10.1007/s12471-020-01367-4

**Published:** 2020-01-24

**Authors:** P. P. T. de Jaegere, A. de Weger, P. den Heijer, M. Verkroost, J. Baan, T. de Kroon, Y. America, G. J. Brandon Bravo Bruinsma

**Affiliations:** 1 Department of Cardiology, Erasmus University, Rotterdam, The Netherlands; 2 Department of Cardiothoracic Surgery, University Hospital Leiden, Leiden, The Netherlands; 3 Department of Cardiology, Amphia Ziekenhuis, Breda, The Netherlands; 4 Department of Cardiothoracic Surgery, University Hospital Nijmegen, Nijmegen, The Netherlands; 5 Department of Cardiology, Academic Medical Centre, Amsterdam, The Netherlands; 6 Department of Cardiothoracic Surgery, Antonius Ziekenhuis Nieuwegein, Nieuwegein, The Netherlands; 7 Department of Cardiology, Rijnstate Ziekenhuis Arnhem, Arnhem, The Netherlands; 8 Department of Cardiothoracic Surgery, Isala Heart Centre, Isala Zwolle, The Netherlands

**Keywords:** Aortic stenosis, Heart team, Surgical aortic valve replacement, Transcatheter aortic valve replacement, Transcatheter aortic valve implantation

## Abstract

The current paper presents a position statement of the Dutch Working Group of Transcatheter Heart Valve Interventions that describes which patients with aortic stenosis should be considered for transcatheter aortic valve implantation and how this treatment proposal/decision should be made. Given the complexity of the disease and the assessment of its severity, in particular in combination with the continuous emergence of new clinical insights and evidence from physiological and randomised clinical studies plus the introduction of novel innovative treatment modalities, the gatekeeper of the treatment proposal/decision and, thus, of qualification for cost reimbursement is the heart team, which consists of dedicated professionals working in specialised centres.

## Introduction

Aortic valve stenosis is a common valvular heart disease, the prevalence of which increases with age, and is associated with a dismal prognosis and quality of life in the presence of symptoms and/or left ventricular (LV) impairment [1]. Survival and quality of life can only be improved by aortic valve replacement, with surgical aortic valve replacement (SAVR) having been the unique and undisputable reference procedure for many decades. Since 2002 a less invasive procedure has been available that consists of the implantation of a trileaflet bioprosthetic valve mounted in a metallic scaffold at the base of the aortic root, predominantly via the common femoral artery, and is termed transcatheter aortic valve implantation (TAVI, Europe) or replacement (TAVR, USA) [2]. In contrast to SAVR, TAVI is a beating heart procedure without the need for general anaesthesia, sterno- or thoracotomy, aortic cross-clamping, cardiac arrest and extracorporeal circulation or other forms of circulatory support [3].

TAVI was conceptualised by Henning R. Andersen (Denmark), whose proposal was not accepted for publication several times, as the topic was considered to have too low a priority. Hence TAVI was first executed in clinical practice by Alain Cribier and co-workers in 2002 (France) [2, 4]. The Netherlands quickly adopted this innovative treatment and played a vital role in further refinement and simplification of TAVI without surgical access and circulatory support (i.e. fully percutaneous TAVI, which experienced its world premiere in October 2006) [5, 6] Thereafter, TAVI was quickly embraced, leading to an explosive increase in its use, outnumbering SAVR procedures and boosting the use of bioprosthetic valves during SAVR even in patients in whom—mainly because of their age—mechanical valves were considered the preferred type [7, 8].

In contrast to percutaneous coronary intervention (PCI), which was first performed in a young male patient with a so-called low-risk coronary artery lesion, TAVI was first applied in a patient who was rejected for SAVR [2]. Clinical practice followed this paradigm and TAVI was considered only in patients who were not candidates for SAVR or at prohibitive risk if undergoing SAVR. Consequently, the first randomised clinical trials that directly compared safety and efficacy of TAVI and SAVR were conducted in this category of patients [9, 10]. Based upon the findings, TAVI became the recommended treatment for elderly patients with aortic stenosis, who are considered not to be candidates for surgery or poor surgical candidates or at increased operative risk for mortality or complications [11, 12].

In the Netherlands there is currently a debate among stakeholders in care—from healthcare providers and their clinical and scientific societies, policy makers and insurance companies to patients and their associated societies—concerning in which patients TAVI should be considered the preferred treatment and, more specifically, whether TAVI in patients with aortic stenosis for whom SAVR involves an intermediate or (more recently) also low risk should constitute medical care that qualifies them for cost reimbursement.

This question is not only of clinical or clinical-scientific interest, it is also highly relevant and even critical for the organisation of care and resource planning both from the perspective of the professionals and their institutions who deliver care (hospitals) and policy makers and affiliated institutions or organisations. For instance, a recent study reported that approximately 42% of patients with symptomatic aortic stenosis do not undergo SAVR and notwithstanding being deemed candidates for TAVI, 38% did not receive TAVI but medical therapy [13]. In addition, the number of patients per annum who are candidates for TAVI in the EU is estimated to be 114,757 (95% confidence interval (CI) 69,380–172,799—Netherlands: 3614 patients (95% CI 2185–548)) when using current indications. This number would increase by 7% to 122,402 (95% CI 38,170–93,322) if all intermediate-risk patients were to receive TAVI. If TAVI became the preferred treatment not only for intermediate-risk patients but also for patients >75 years at low surgical risk, there would be a further 50% increase of TAVI candidates to 177,462 per annum (95% CI 110,059–260,576; Netherlands: 5524 patients (95% CI 3467–8126)).

Given the above, the concern of the Dutch Healthcare Authorities and the question as to whether the cost of TAVI should be reimbursed in patients with aortic stenosis for whom SAVR involves an intermediate risk is understandable.

## Clinical-scientific evidence in ‘intermediate-risk’ patients

The best evidence available to answer the question are the results and conclusions of four landmark randomised controlled trials (RCTs) that directly compared TAVI with SAVR in patients at intermediate and low surgical risk [14–18]. The studies and their results are summarised in Tab. 1, 2 and 3. Considering the primary endpoint, the conclusion is that TAVI is non-inferior to SAVR and superior when transfemoral access is possible in patients at intermediate and low surgical risk. In addition, a different pattern of other adverse outcomes is observed with TAVI than with SAVR, such as a higher incidence of permanent pacemaker implantation after TAVI (especially when using self-expanding valves) but a higher incidence of bleeding, acute kidney insufficiency and new-onset atrial fibrillation after SAVR (Tab. 2 and 3).

**Table 1:** Landmark studies [14–18] comparing transcatheter aortic valve implantation (*TAVI*) with surgical aortic valve replacement (*SAVR*) in patients with aortic stenosis at intermediate (PARTNER 2A, SURTAVI) and low risk (PARTNER 3, Evolut) if undergoing SAVR

	PARTNER 2A	SURTAVI	Thourani et al	Partner 3	Evolut
Publication	2016	2016	2017	2019	2019
Design	RCT	RCT	Propensity matched analysis	RCT	RCT
Hypothesis	TAVI non-inferior	TAVI non-inferior	n.a.	TAVI non-inferior	TAVI non-inferior
Primary endpoint	All-cause death or disabling stroke at 2 years	All-cause death or disabling stroke at 2 years	All-cause death, stroke, ≥moderate PVL at 1 year	All-cause death, stroke, or rehospitalisation at 1 year	All-cause death or disabling stroke at 2 years
Risk assessment	By heart team (guided by STS 4–8)	By heart team (guided by STS 3–15)	By heart team (guided by STS >4)	By heart team (guided by STS <4)	By heart team (guided by STS <3)
No. of patients	2032	1746	2021	1000	1468

*PVL* paravalvular leak, *RCT* randomised controlled study, *STS* Society of Thoracic Surgery risk score

**Table 2:** Components of outcomes, landmark studies [14–18] comparing transcatheter aortic valve implantation (*TAVI*) with surgical aortic valve replacement (*SAVR*) in patients with aortic stenosis at intermediate (**bold**) and low risk (*italic*) if undergoing SAVR

	PARTNER 2A		SURTAVI		Thourani et al		PARTNER 3		Evolut	
	TAVI	SAVR	TAVI	SAVR	TAVI	SAVR	TAVI	SAVR	TAVI	SAVR
Age	**81.5 (6.7)**	**81.7 (6.7)**	**79.9 (6.2)**	**79.8 (6.0)**	**81.9 (6.6)**	**81.6 (6.8)**	*73 (6)*	*73 (6)*	*74 (6)*	*74 (6)*
STS score	** 5.8 (2.1)**	** 5.8 (2.1)**	** 4.4 (1.5)**	** 4.5 (1.6)**	** 5.2 (4.3–6.3)**	** 5.4 (4.4–6.7)**	* 1.9 (0.7)*	* 1.9 (0.6)*	* 1.9 (0.7)*	* 1.9 (0.7)*
Death^a^	** 3.4**	** 4.0**	** 2.2**	** 1.7**	** 1.1**	** 4.0**	* 0.4*	* 1.1*	* 0.5*	* 1.3*
Death at FU	**16.3**	**17.9**	**11.4**	**11.6**	** 7.4**	**13.0**	* 1.0*	* 2.5*	* 2.4*	* 3.0*
All stroke^a^	** 6.5**	** 6.5**	** 3.4**	** 5.6**	** 2.7**	** 6.1**	* 0.6*	* 2.4*	* 3.4*	* 3.4*
All stroke at FU	**12.8**	**11.1**	** 6.2**	** 8.4**	** 4.6**	** 8.2**	* 1.2*	* 3.1*	* 4.1*	* 4.3*
Disabling stroke^a^	** 3.2**	** 4.4**	** 1.2**	** 2.5**	** 1.0**	** 4.4**	* 0.0*	* 0.4*	* 0.5*	* 1.7*
Disabling stroke at FU	** 6.2**	** 6.5**	** 2.6**	** 4.5**	** 2.3**	** 5.9**	* 0.2*	* 0.9*	* 0.8*	* 2.4*
Major bleeding^a^	**10.5**	**46.9**	**12.2**	** 9.3**	** 4.6**	**46.7**	* 3.6*	*24.5*	* 2.4*	* 7.5*
Bleeding at FU	**17.3**	**50.2**	**nr**	**nr**	**nr**	**nr**	* 7.7*	*25.9*	* 3.2*	* 8.9*
AKI ^a^	** 1.2**	** 3.3**	**nr**	**nr**	** 0.5**	** 3.3**	* 0.4*	* 1.8*	* 0.9*	* 2.8*
AKI at FU	** 3.8**	** 6.4**	**nr**	**nr**	**nr**	**nr**	*nr*	*nr*	* 0.9*	* 2.8*
New-onset A. FIB^a^	** 9.1**	**28.3**	**12.9**	**43.4**	** 5.0**	**28.3**	* 5.0*	*39.5*	* 7.7*	*35.4*
New A. FIB at FU	**11.3**	**29.3**	**nr**	**nr**	** 5.9**	**29.2**	* 7.0*	*40.9*	* 9.8*	*38.3*
PPI*	** 8.6**	** 7.3**	**25.9**	** 6.6**	**10.2**	** 7.3**	* 6.6*	* 4.1*	*17.4*	* 6.1*
PPI at FU	**12.0**	**10.8**	**nr**	**nr**	**12.4**	** 9.4**	* 7.5*	* 5.5*	*19.4*	* 6.7*

Age and STS are expressed using either mean (±SD) or median (IQR)

Outcomes are expressed as relative number

*AKI* acute kidney failure, *A. FIB* atrial fibrillation, *FU* follow-up, *nr* not reported, *PPI* permanent pacemaker implantation, *STS* Society of Thoracic Surgery

^a^Clinical endpoint at 30 days

**Table 3:** Primary endpoint of landmark studies [14–18] comparing transcatheter aortic valve implantation (*TAVI*) with surgical aortic valve replacement (*SAVR*) in patients with aortic stenosis at intermediate and low risk if undergoing SAVR

**PARTNER 2A**			
	*All TAVI patients*	*SAVR*	*Hazard ratio (95% CI)*
Intention to treat	19.3	21.1	0.89 (0.73–1.0)
As treated	18.9	21.0	0.87 (0.71–1.07)
	*Transfemoral TAVI patients*	*SAVR*	*–*
Intention to treat	16.8	20.4	0.79 (0.62–1.00)
As treated	16.3	20.0	0.78 (0.61–0.99)
**SURTAVI**			
	*All TAVI patients*	*SAVR*	*∆ (95% CI)*
Intention to treat	12.6	14.0	−1.4 (−5.2 to 2.3)
As treated	–	–	–
**PARTNER 3**			
	*Transfemoral TAVI (= all patients)*	*SAVR*	*–*
As treated	8.5	15.1	−6.6 (−10.8 to −2.5)
**Evolut**			
	*All TAVI patients*	*SAVR*	*∆ (95% CI)*
As treated	5.3	6.7	−1.4 (−4.9 to 2.1)

Primary endpoint is expressed as relative number. *CI* confidence interval

**Table 4:** Components of primary endpoint of propensity-matched analysis comparing transcatheter aortic valve implantation (*TAVI*) with surgical aortic valve replacement (*SAVR*) in patients with aortic stenosis at intermediate risk if undergoing SAVR

	All TAVI patients	SAVR	Transfemoral TAVI	SAVR
Death	7.4	13.0	6.5	12.2
Stroke	4.6	8.2	4.3	8.6
≥moderate PVL	1.5	0.4	nr	nr

*nr* not reported, *PVL* paravalvular leak

Thourani et al. used the PARTNER 2 observational and PARTNER 2A randomised trial to compare outcome (follow-up duration 12 months) between TAVI and SAVR in intermediate-risk patients [16]. To correct for confounding in the statistical comparison—due to imbalance in baseline characteristics—propensity scores were calculated for each individual patient representing the likelihood of being treated with TAVI followed by partitioning into five quintiles: 1, lowest 20% likelihood; 5, highest 20% likelihood. In each quintile, patients undergoing TAVI had a lower incidence of the composite primary endpoint than patients who underwent SAVR (ranging from −14.5% in quintile 1 to −4.3% in quintile 5). Moreover, TAVI was superior to SAVR for the composite endpoint (weighted difference of proportions −9.2%, 95% CI −13.0 to −5.4; *p* < 0.0001) and for the individual outcomes of death (−5.2%, −8.0 to −2.4; *p* = 0.0003) and stroke (−3.5%, −5.9 to −1.1; *p* = 0.0038). SAVR was superior to TAVI for ≥moderate aortic regurgitation (1.2%, 0.2–2.2; *p* = 0.01). Based on their findings the authors conclude that ‘TAVR should be considered as the preferred alternative to SAVR in intermediate-risk patients and an expected extension of indication to the surgical candidate in the near future’.

## A different approach from scientific societies and medical professionals

This committee believes, however, that the question of the Dutch Healthcare Authorities should be rephrased more generically, namely as ‘Which patients with aortic stenosis should be considered for TAVI and how should treatment decisions be made?’ The reasons for reformulating the question are elaborated in the following paragraphs, which address a number of issues related to the complexity of medical decision-making in the individual patient and the fast and incessant technological evolution and innovations in aortic valve replacement therapy affecting clinical medicine, followed by the conclusions and recommendations of the Dutch Working Group of Transcatheter Heart Interventions.

### Risk models—can we define risk in the individual patient?

The medical, medical-scientific and non-medical communities use a risk score or a risk score model to categorise patients into various risk categories on the basis of an arbitrary cut-off value. Various cut-off values are used. In the landmark RCTs, the Society of Thoracic Surgery (STS) risk score (risk of death at 30 days after surgery) was used [14–17]. For the definition of intermediate risk, the PARTNER 2A study used a cut-off value of STS ≥4–8% while the SURTAVI trial used a margin of 3–15%. Low risk was defined by STS <3 or 4 [16, 17]. The European Society of Cardiology (ESC) left the original three-category system (high, intermediate and low risk) and proposes to break down patients into low or non-low risk. Low risk is defined by a STS or EuroSCORE 1 score <4% or a logistic EuroSCORE <10%. Non-low risk is consequently defined by a STS or EuroSCORE 1 score of ≥4 or a logistic EuroSCORE ≥10%.

Interestingly, neither the STS nor the Euro- or logistic EuroSCORE has been designed for patients undergoing TAVI but for patients undergoing open-heart surgery in general, while a model performs best when developed from a population that undergoes the specific procedure. The use of both the STS and the EuroSCORE in patients referred for TAVI has, therefore, been questioned openly. There are currently two TAVI-specific risk scores, the FRANCE‑2 model and the Risk Prediction model proposed by the STS/American College of Cardiology (ACC) Transcatheter Valve Therapy (TVT) Registry [19, 20]. The latter is the most robust and sound one and consists of a development and validation model. The development model is based upon a series of 13,718 out of 13,887 patients (only 1.2% patients with missing data) who received TAVI outside the landmark RCTs between 2011 and 2014 at 265 sites in the USA. The validation model is based upon 6868 out of 6877 patients (only 9 patients with missing data) who received TAVI in 2014 at 314 sites. At variance with the STS and EuroSCORE, the endpoint or outcome was mortality at discharge, which was chosen as such given its clinical relevance, easiness to define and collect. The development task force of the TVT Registry selected an initial set of covariates that was reduced to 9 after statistical analysis and TVT Registry Steering Committee evaluation. Considering the limitations (i.e. single short-term endpoint, no inclusion of prediction of neurological deficit, exclusion of covariates that also affect the primary endpoint, such as frailty) the main finding was that the STS/ACC TVT risk model showed excellent calibration in the overall number of patients undergoing TAVI and in various clinical subsets. Also, the STS/ACC TVT model performed better than the STS and EuroSCORE models. Given the limitations of the current model, the investigators are continuing to refine and improve the model by including the prediction of 1‑year mortality in addition to non-fatal outcomes and the inclusion of more covariates so as to offer an instrument to help physicians in the delivery of patient-centred care.

Nevertheless, it is acknowledged by the experts and medical professionals that any risk model will remain a distant abstract of the complex reality of clinical decision-making, as no model will be able to include all covariates affecting mortality and non-fatal complications during and shortly after cardiac interventions and will always fall short as regards accurately predicting the risk in the individual patient. For that reason, the medical community and their scientific societies both in the EU and USA emphasise that a model should not indicate which patient is a candidate for TAVI but to be considered and used as a single element in the selection process in concert with all the other information collected by the physician when he/she sees and examines the patient (clinical judgement based upon a cardiac-specific and general history and physical examination) in addition to technical assessments such as laboratory (e.g. haemoglobin, kidney function, NT-proBNP …) and cardiovascular imaging [11, 12]. The deficiencies of risk models were recognised during the design phase of the landmark RCTs and are the reason why the heart team received a central and dominant role in the process of patient selection for aortic valve replacement therapy [14–17].

### The heart team: defining indication and optimal treatment modality in the individual patient

To address the deficiencies of risk models, the medical-scientific organisations in both the EU and USA strongly endorse the role of the heart team in making the treatment decision [12]. The update of the 2017 European and American guidelines explicitly states that ‘the core principle in the ESC approach is to emphasise a complex decision process with a list of specific factors to be considered in each patient, rather than defining broad patient groups that should undergo TAVI or SAVR’ [12]. The guidelines first of all state that aortic valve interventions should be performed only in centres with both departments of cardiology and cardiac surgery on site and with structured collaboration between the two, including a heart team. The choice of the intervention must be based upon careful individual evaluation of technical suitability and weighing-up of the risks and benefits of each modality. These should be discussed with the patient and their family in accordance with the policy of the healthcare authorities to involve the patient and family in the decision-making process, as discussed below. In addition, the local expertise and outcomes for the given intervention must be taken into account. The 2017 ESC guidelines help the physician and heart team by providing a list of variables to be considered, many of which are not included as covariates in the current risk prediction models [11, 12].

The concept of the heart team and, thus, a collaborative approach between interventional cardiologists and cardiothoracic surgeons to jointly deliver appropriate care in a timely manner while minimising unnecessary investigations and procedures was recognised in the early 2000s for patients with (complex) cardiac disease. In the resulting joint publications of the scientific societies (cardiology, cardiothoracic surgery) in both the EU and USA, recommendations were provided for the delivery and organisation of care in 2006 and 2010 [21].

The dominant role of the heart team is confirmed by the ESC Working Group on Valvular Heart Disease, which goes one step further by recommending the creation of Heart Valve Clinics (HVC), in which a group of healthcare professionals with expertise in heart valve disease (HVD) work in a dedicated environment to provide specialised and centralised evaluation, care and education to patients with HVD [21]. The reason is the observed variability in the degree of expertise in the management of patients with HVD associated with inappropriate use of care and facilities (i.e. under- or overtreatment) due to—among other factors—the complexity of establishing the correct diagnosis (severity of the HVD in particular) and deciding on the correct timing and type of intervention in a patient with VHD. The objective of creating HVC is to improve the appropriateness of care (diagnosis, treatment decision, follow-up) in addition to training and education of patients and medical professionals.

In accordance with the updated 2017 European and American guidelines, this committee sees the role of the heart team as follows:

1. Verification of the correctness of the diagnosis and the indication for the intervention,
2. Treatment proposal; valve replacement or medical therapy with
  - written documentation of the motivation for the treatment proposal in the case of medical therapy and
  - in the case of valve replacement therapy, written documentation of the motivation for the proposed mode of valve replacement therapy (i.e. catheter-based or surgical) and the preferred valve type (bioprosthetic or mechanical).

#### Verification of correctness of diagnosis and indication

For this purpose, the presence of an imaging specialist during the heart team meeting is essential [11, 12, 21]. His/her presence is particularly important given the intricacies in not so much the verification of the presence and aetiology of disease but especially its severity and effects on the heart and cardiovascular system. The adagio of Catherine Otto, ‘Listen to the patient and look at the valve’ or ‘Treat the patient and not the number’, remains very true, most likely for many years to come, despite improved understanding of the pathophysiology of the disease process and its consequences [22, 23]. The effect of LV geometry and function in the assessment of the severity of aortic stenosis is well understood and widely recognised [24]. This is not the case for the presence of systemic hypertension or other factors affecting ventriculo-arterial coupling [25]. Another ongoing debate is whether asymptomatic patients with severe aortic stenosis and preserved LV function should be denied aortic valve replacement therapy [26]. This also holds for patients with moderately severe aortic stenosis and impaired LV function.

In clinical practice, cut-off values help to discern non-severe and severe aortic stenosis and, thus, the treatment indication. While on a population level these cut-off values (e.g. antegrade velocity, valve area) may be useful to differentiate patient categories at increased risk of death, they are less useful in the individual patient due to overlap of these cut-off values in the various risk groups and—similar to the assessment of the severity of mitral regurgitation—the dynamic interaction between the valve, left ventricular and arterial system when measuring these values. It reinforces the role of the heart team and HVC in the treatment decision (and selection) by providing an expert collaborative (medical specialities) and integrative approach (patient symptoms, antecedents, co-morbidity, valve anatomy, function and consequences—electrocardiography, echocardiography, laboratory data).

The presence of a geriatrician is recommended particularly when a patient ≥75 years or younger but with co-morbid conditions that raise the suspicion of neurocognitive dysfunction and/or frailty is discussed. It is recommended that such patients are first seen by the geriatrician before the heart team meeting and certainly before the final treatment decision so as to avoid the treatment being futile.

#### Treatment proposal

The ESC guidelines are to be used as a framework for decision-making but, as pointed out by the ESC Guideline Committee, the decision should be individualised to each patient’s situation [11, 12]. This committee recognises that medical decision-making is changing, with increasing involvement of patient and family, which is in line with the endorsement of the development of more patient-centred care promoted by healthcare authorities in both the EU and USA [27]. As stated by the Food and Drug Administration (FDA), individual patients may experience the effects of disease and therapies differently and may have a unique perspective about diagnostic procedures or treatments that differ from those of other patients or their healthcare provider. This is reason why the FDA and the European Medicines Agency (EMA) created the FDA/EMA Patient Engagement Cluster, which is a working group on the importance and role of patient engagement. For that purpose, the patient-reported outcome is now being incorporated in the clinical-scientific evaluation in addition to the standard measures of safety and efficacy, as it measures the patient’s response concerning his/her health condition without caregiver interpretation.

#### Valve type

Valve type selection should be based upon balancing the risk of bleeding associated with oral anticoagulant therapy when choosing a mechanical valve and the risk of structural valve deterioration and the need for a redo operation or intervention when choosing a bioprosthetic valve. Both the ESC and American Heart Association (AHA) guidelines stress this should be a shared decision-making process in which the patient’s age, expected longevity and preferences need to be included (class 1 recommendation). In general, they recommend a mechanical valve in patients aged under 50 or 60 and a bioprosthetic valve in patients aged over 65 or 70 years [12]. It has to be recognised that valve replacement therapy is the substitution of a disease with a more benign condition mandating careful instruction and information of the patient and structured follow-up. Bioprosthetic valves are increasingly being used in younger patients who—by virtue of their age—are at higher risk of structural valve deterioration [28]. Yet, the use of bileaflet mechanical valves is associated with a considerable lifetime risk of valve-related morbidity such as thromboembolism (18%), bleeding (15%) and reintervention (10%) [29]. Hypo-attenuated leaflet thickening and early-reduced leaflet motion have been reported in approximately 10% of the patients who underwent advanced imaging after bioprosthetic valve implantation (TAVI) and replacement (SAVR) [30]. Their clinical significance remains to be elucidated but they are assumed to be markers of a thrombotic process triggered by the valve replacement procedure. This matter is the subject of a number of RCTs evaluating optimal antithrombotic therapy after bioprosthetic valve implantation [30].

The above highlights the complexity of current medical decision-making. The role of the heart team is to make a balanced decision after careful weighing-up of the risks and benefits of catheter-based and surgical aortic valve replacement as suggested by the ESC guidelines and the ESC Working Group on Valvular Heart Disease and following the recommendation of healthcare policies regarding patient involvement. This committee, therefore, strongly recommends that the motivation for the treatment proposal made by the heart team is collected in the electronic patient dossier for reasons of auditing and verification both for clinical and clinical-scientific reasons, and should be accompanied by a shared-decision agreement with the patient, after a careful discussion on the topic.

## Data base: to adequately assess risks and benefit of proposed treatment modalities—institutional performance

Since the heart team needs to weigh up the risk and benefits of the proposed treatment modality, the local heart team must be informed about the outcome of the proposed treatment in the local institution. This implies a local infrastructure and organisation of data collection that is capable of providing valid monitoring and evaluation of care as proposed by the ESC Working Group on Valvular Heart Disease [21]. In concordance, participation in a nationally audited registry—in addition to written documentation of the motivation for the heart team’s treatment proposal—should be the prerequisite for reimbursement of the care delivered (i.e. medical treatment, catheter-based or surgical valve replacement therapy).

The objective of the nationwide registry is to monitor and evaluate the care of patients with VHD referred to hospitals certified for valvular heart replacement therapy in the Netherlands in addition to the evolution of care. This implies essentially:

1. definition of a minimal data set that captures the indispensable data of the patient, procedure and initial follow-up (e.g. at hospital discharge or at 30 days or whichever comes first) to monitor and evaluate the outcome and evolution of care,
2. definition of the responsibilities of the persons involved in the organisation and management of the nationwide data base.

While Europe was the cradle of TAVI, the USA took the lead in organising and conducting the landmark trials that have affected and still greatly affect the management of patients with VHD (aortic stenosis in particular) and the creation of a nationwide data registration system for:

- evaluation of the results of the landmark RCTs (added value of registry data on outcome in relation to those of patient populations recruited in the framework of RCTs in expert cardiac centres),
- post-market surveillance (an FDA prerequisite),
- covering of care (national coverage decision by the US Centers of Medicare and Medicaid Services (CMS)).

The EU, and the Netherlands in particular, must follow the mature and professional way in which the nationwide registry has been organised in the USA. The US professional medical societies (the Society of Thoracic Surgeons (STS) and the American College of Cardiology (ACC)) developed the Transcatheter Valve Therapy (TVT) Registry in collaboration with the FDA, CMS and valve manufacturers [31]. The definition of the data set was based upon the focus of collecting those data that are critically important to adequately measure the quality of care:

- TVT Registry hospital participants capture data but these are collected and stored by the ACC. On a quarterly basis the ACC transmits the data to an independent research institute (Duke Clinical Research Institute, DCRI).
- Outcomes are adjudicated by a board-certified cardiologist at the DCRI using Valve Academic Research Consortium‑2 (VARC-2) definitions.
- The DCRI performs analyses for research and publications.
- A Research and Publication Committee has been established to evaluate and approve research proposals and to monitor their progress.
- A Registry Steering Committee overseeing the strategic direction of the registry is composed of physicians appointed from the STS and ACC, FDA and CMS representatives, and staff members of STS and ACC.
- A Stakeholder Advisory Committee includes members from device manufacturers, patients, consumers, FDA and CMS, physicians and health systems, and provides meaningful input regarding the needs and goals of each of the stakeholders.

Such a professional and complete structure and infrastructure that guarantees the quality of the data set in addition to relevant outcome reports is essential. The Dutch Society of Cardiology and the Society of Cardiothoracic Surgery endorse this initiative, which is currently taking place in the Netherlands.

## Conclusion/summary

There is currently debate in the Netherlands as to whether TAVI for patients with aortic stenosis for whom SAVR would involve an intermediate or currently also low risk should be considered for reimbursement. This committee believes that instead of using arbitrarily defined quantitative cut-off values to categorise patients into various risk groups, reimbursement of the cost of care should be based upon the appropriateness of care. Given the complexity of the disease in terms of diagnosis, treatment decision, treatment execution and evaluation, this is best done by a team of experts in VHD (heart team) working in dedicated centres that qualify for the treatment of VHD. As elaborated in this article, categorisation of patients into risk groups falls short when it comes to the evaluation of the individual patient. Also, the rapid technological evolution of minimally invasive treatment of VHD will change the management of patients with VHD, such as asymptomatic patients or patients with moderate aortic stenosis and impaired LV functions. Further developments in indications may be expected as treatment becomes less and less invasive while maintaining and even improving the current outcome measures of safety and efficacy (e.g. early intervention with the objective of preserving LV function by avoiding the development of myocardial fibrosis). Given this outlook, the role of the expert heart team and heart valve centres is and will be even more crucial to guarantee the appropriateness of care, treatment decision and modality in particular. It obviates the need for repetitive reevaluation of whether a novel treatment modality or patient category meets the requirements of reimbursement of the costs of treatment.

In accordance with the medical-clinical and clinical-scientific societies of cardiology and cardiothoracic surgery in both the EU and USA, the Dutch Working Group of Transcatheter Heart Interventions recommends that the Dutch Healthcare Authorities provide coverage of every patient with aortic stenosis who will be or has been treated with TAVI, provided:

- The dossier of the patient has been discussed in the multidisciplinary heart team meeting of an institution that qualifies to perform transcatheter heart interventions in accordance with the Competence Document of the Dutch Society of Cardiology (NVVC) and the Dutch Society of Thoracic Surgery (NvT).
- The treatment decision and modality are based upon the Indication Document of the NVVC and NvT (appendix 2—a document that is annually revised and updated if necessary by the Dutch Working Group of Transcatheter Heart Interventions on the basis of clinical evidence published in peer-reviewed journals and guidelines of scientific societies) which are captured in the electronic patient file, including the motivation for these, in such a way that treatment decision, modality and motivation are suitable for retrospective verification for internal and external auditing.
- The institution that performs transcatheter heart interventions participates in a nationwide prospective registry that—for reasons of independence and quality—complies with the organisational infrastructure and set-up of the ACC/AHA TVT Registry.
